# Which Dimensions of Patient-Centeredness Matter? - Results of a Web-Based Expert Delphi Survey

**DOI:** 10.1371/journal.pone.0141978

**Published:** 2015-11-05

**Authors:** Jördis M. Zill, Isabelle Scholl, Martin Härter, Jörg Dirmaier

**Affiliations:** Department of Medical Psychology, University Medical Center Hamburg-Eppendorf, Hamburg, Germany; Providence VA Medical Center and Brown University, UNITED STATES

## Abstract

**Background:**

Present models and definitions of patient-centeredness revealed a lack of conceptual clarity. Based on a prior systematic literature review, we developed an integrative model with 15 dimensions of patient-centeredness. The aims of this study were to 1) validate, and 2) prioritize these dimensions.

**Method:**

A two-round web-based Delphi study was conducted. 297 international experts were invited to participate. In round one they were asked to 1) give an individual rating on a nine-point-scale on relevance and clarity of the dimensions, 2) add missing dimensions, and 3) prioritize the dimensions. In round two, experts received feedback about the results of round one and were asked to reflect and re-rate their own results. The cut-off for the validation of a dimension was a median < 7 on one of the criteria.

**Results:**

105 experts participated in round one and 71 in round two. In round one, one new dimension was suggested and included for discussion in round two. In round two, this dimension did not reach sufficient ratings to be included in the model. Eleven dimensions reached a median ≥ 7 on both criteria (relevance and clarity). Four dimensions had a median < 7 on one or both criteria. The five dimensions rated as most important were: *patient as a unique person*, *patient involvement in care*, *patient information*, *clinician-patient communication* and *patient empowerment*.

**Discussion:**

11 out of the 15 dimensions have been validated through experts’ ratings. Further research on the four dimensions that received insufficient ratings is recommended. The priority order of the dimensions can help researchers and clinicians to focus on the most important dimensions of patient-centeredness. Overall, the model provides a useful framework that can be used in the development of measures, interventions, and medical education curricula, as well as the adoption of a new perspective in health policy.

## Background

### Introduction

Patient-centeredness is an internationally widely discussed topic in high-quality health care and modern medicine [1–5]. Moreover, there is an increased recognition of the concept at the health policy level. Already in 2001, the US Institute of Medicine’s (IOM) publication “Crossing the Quality Chasm: A New Health System for the 21^st^ Century” declared patient-centeredness to be one of six goals for health care improvement of the US health care system [6, 7]. In 2003, Health Canada highlighted the essential meaning of patient-centeredness in health care by creating the initiative Interdisciplinary Education for Collaborative Patient-Centered Practice (IECPCP) [8]. In Australia, patient-centered care is supported by the Australian Charter of Healthcare Rights, which ensures the provision of safe and high-quality care and by the National Safety and Quality Framework for health care, which was developed in 2009 to improve the healthcare system [9]. In the UK, patient-centeredness is a core concept in professional medical guidance [10]. In Germany, a large research priority program on patient-centeredness and chronic diseases was launched from the government in 2007, including a total funding volume of over 20 million Euro allocated to 77 research projects (www.research-patientcenteredcare.org).

Despite a large amount of research addressing patient-centeredness, no global consensus regarding its definition and conceptualization has been established yet. Van Dulmen [11] states that it is a “fuzzy concept”, which lead to more confusion than clarity. Moreover, Epstein and colleagues [12] outline it as a “multifaceted construct, like intelligence”, and Hobbs [13] describes patient-centeredness as a “poorly conceptualized phenomenon”. Similar results about the ambiguity of the German term for patient-centeredness were found in a pilot study of this project [14].

Accordingly, existing definitions of the term differ considerably. Gerteis et al. [15] identified seven domains of patient-centered care (e.g. respect for patients’ values, preferences, and needs; co-ordination and integration of care). The dimensions of Gerteis et al. were adopted by the Picker Institute in 2004 and have been used since then to evaluate patient-centeredness in the US and Europe [16]. The IOM defined the concept as “care that is respectful of and responsive to individual patient preferences, needs and values and ensuring that patients’ values guide all clinical decisions” [6]. Stewart et al. postulated six interactive elements of the patient-centered method (e.g. exploring both the disease and the illness experience, understanding the whole person) [17, 18]. Epstein [12] described patient-centered communication in four domains of (e.g., eliciting and understanding the patient’s perspective and concerns, helping patients to share power and responsibility). Another widely cited concept was developed by Mead and Bower, who derived five key dimensions of patient-centeredness from a literature review (e.g. sharing power and responsibility, therapeutic alliance) [19, 20].

Some recent research concentrated on the benefits of patient-centered care. For instance, a systematic review of over 40 studies conducted by Rathert et al. (2012) examined the relationship between dimensions of patient-centered care and patient satisfaction, patient clinical and organizational outcomes [21]. Overall, the review showed that patient-centeredness had positive effects on these outcomes. Nevertheless, since definitions and therefore measurement of patient-centeredness are very heterogeneous, the comparability of research results on patient-centeredness is questionable.

In a previous step of this study, which was part of a larger project on the evaluation of dimensions and measurement scales in patient-centeredness [22], we conducted a systematic review to identify different dimensions and definitions of patient-centeredness described in the literature. In this initial review we screened all eligible references whether they contained a conceptual definition of patient-centeredness in the full text. If they fulfilled this inclusion criterion, they were included in the subsequent content analysis. The definitions of patient-centeredness described in these full texts were coded. Finally, the codes were aggregated into different dimensions of patient-centeredness and then summarized into an integrative model [23]. The following 15 dimensions of patient-centeredness were identified: *essential characteristics of the clinician*, *clinician-patient relationship*, *clinician-patient communication*, *patient as a unique person*, *biopsychosocial perspective*, *patient information*, *patient involvement in care*, *involvement of family and friends*, *patient empowerment*, *physical support*, *emotional support*, *access to care*, *integration of medical and non-medical care*, *coordination and continuity of care*, *teamwork and teambuilding*. A short description of the identified dimensions is displayed in Table 1. The dimensions were categorized as enablers, principles, or activities of patient-centered care. A detailed descriptions of the dimensions and the model can be found in the original publication [23].

**Table 1:** Dimensions of patient-centeredness [23].

Dimension	Brief description
Principles	
Essential characteristics of the clinician	A set of attitudes towards the patient (e.g. empathy, respect, honesty) and oneself (self-reflectiveness) as well as medical competency
Clinician-patient relationship	A partnership with the patient that is characterized by trust and caring
Patient as a unique person	Recognition of each patient’s uniqueness (individual needs, preferences, values, feelings, beliefs, concerns and ideas, and expectations)
Biopsychosocial perspective	Recognition of the patient as a whole person in his or her biological, psychological, and social context
Enablers	
Clinician-patient communication	A set of verbal and nonverbal communication skills
Integration of medical and non-medical care	Recognition and integration of non-medical aspects of care (e.g. patient support services) into health care services
Teamwork and teambuilding	Recognition of the importance of effective teams characterized by a set of qualities (e.g. respect, trust, shared responsibilities, values, and visions) and facilitation of the development of such teams
Access to care	Facilitation of timely access to healthcare that is tailored to the patient (e.g. decentralized services)
Coordination and continuity of care	Facilitation of healthcare that is well coordinated (e.g. regarding follow-up arrangements) and allows continuity (e.g. a well-working transition of care from inpatient to outpatient)
Activities	
Patient information	Provision of tailored information while taking into account the patient’s information needs and preferences
Patient involvement in care	Active involvement of and collaboration with the patient regarding decisions related to the patient’s health while taking into account the patient’s preference for involvement
Involvement of family and friends	Active involvement of and support for the patient’s relatives and friends to the degree that the patient prefers
Patient empowerment	Recognition and active support of the patient’s ability and responsibility to self-manage his or her disease
Physical support	A set of behavior that ensures physical support for the patient (e.g. pain management, assistance with daily living needs)
Emotional support	Recognition of the patient’s emotional state and a set of behavior that ensures emotional support for the patient

Since a clear definition of patient-centeredness is the first step to its successful implementation in routine care, the aims of the present study were:

1. to validate the dimensions of the model derived from the systematic review; and
2. to prioritize the identified dimensions of patient-centeredness.

## Methods

### Ethical considerations

The local Ethics Committee of the Medical Association Hamburg (Germany) was consulted for ethical approval. They informed us that ethical approval was not necessary because no patients were enrolled in the study. Nevertheless, ethical principles were followed throughout the examination and the study was carried out in accordance with the Code of Ethics of the Declaration of Helsinki. The experts were informed on the data collection and analysis. Participation was on a voluntary basis, and data protection rules were considered.

### The Delphi Method

We conducted a Delphi survey, as it is the method of choice for a structured group discussion technique with the aim to reach a high group consensus [24]. This method is widely used, e.g. in the development of clinical practice guidelines [25] and healthcare quality indicators [26]. The Delphi method employs a series of rounds to collect information from and transmit information to participants [26]. A web-based Delphi survey has the advantage of enabling individuals from different locations to converge anonymously and discuss their expert opinions [26]. The procedure mainly followed the recommendations proposed by Boulkedid et al. (2011) [26].

In this study, a two-step web-based rating process was used to reach consensus and prioritize the dimensions of patient-centeredness in terms of their importance and to rate each dimension separately on the criteria ***relevance*** and ***clarity***. The Delphi survey was conducted between February and April 2013. The survey was structured in two rounds and international experts (clinicians, patient representatives, researchers and quality managers) on this topic were invited to participate. In the first round, the experts were asked to rate these dimensions individually, and in the second round they were asked to reflect and rerate their results from the first round.

Since there are no clear recommendations for the validation of a model using a Delphi procedure, we decided, inspired by the procedure of Uphoff and colleagues [27], that a dimension was sufficiently relevant and/or clear if it reached a median ≥ 7 and a consensus ≥ 50% on a tertile seven to nine on a Likert-scale on the criteria of relevance and/or clarity.

### Participants of the expert panel

We aimed to include a broad range of experts in the field of patient-centered care. We therefore invited clinicians, patient representatives, researchers and quality managers from different countries to participate in this study. They were recruited through several strategies: a) identification and invitation of key authors on patient-centeredness, b) identification and invitation of representatives of institutions associated with the field of patient-centeredness, and c) through personal knowledge (e.g. collaboration partners). The survey was available in German and English.

### Step1: Individual rating and prioritizing of the dimensions by the expert panel

In the initial step of the Delphi survey, the selected experts were invited via email and received a link to the survey. The web-based survey contained a list of the 15 dimensions of patient-centeredness identified in the literature review, and a short description of each dimension [23]. Every panel member was instructed to rate the dimensions on a nine-point scale on the criteria of ***relevance*** and ***clarity*** (1 = not relevant/ not clear to 9 = very relevant/ very clear), (e.g. *To what extent would you consider the dimension "Patient as a unique person" relevant/clear*?). Furthermore, they were invited to prioritize the five dimensions they considered most important out of the 15 dimensions (*Please rate the five most important dimensions in your understanding of patient-centeredness)*. Finally, the experts were asked to add, if necessary, further dimensions of patient-centeredness, which were not included so far (*If you think any dimensions in your understanding of patient-centeredness are missing in our identification*, *please name them here)*. Additionally, the survey included questions on demographic and professional characteristics of the experts. The experts were also asked to rate how well informed they consider themselves to be in the field of patient-centeredness, using a five-point response scale ranging from 1 = not at all well to 5 = very well.

For the completion of the first round of the Delphi survey, a timeframe of three weeks was set. Invited experts received one email reminder after two weeks.

The findings of the first round of the Delphi survey were analyzed using the statistical software PASW Statistics (Version 18.0) and summarized in individualized reports to inform the second round of the survey. These reports included the results (median, maximum and minimum) of the panel’s rating in the first round and the individual rating of the respective panel member. It also included an overview of the newly proposed dimensions, in order to allow them to be rated regarding clarity and relevance in the second round.

### Step 2: Rerating and validating of the dimensions by the expert panel

Three weeks after the timeframe of the first round was finished, the panel members who had participated in the first round, received an email with a link to the second round of the web-based Delphi survey. The above described individualized reports were presented in this second survey to the respective panel members with the invitation to compare their individual ratings to the results of the other panel members. They were instructed to adjust or confirm their initial rating and prioritization of the dimensions when deemed necessary. Furthermore, they were asked to rate the newly proposed dimensions regarding their relevance and clarity (similar to the first round). Finally, the ratings of the subgroups of different stakeholders were analyzed separately using the statistical software PASW Statistics (Version 18.0) for the results of the second round. Differences of at least 1.5 points on the rating scale were considered relevant. Significance testing was planned, if subgroups reached a sample size of n ≥ 30.

For the completion of the second round of the Delphi survey, a timeframe of three weeks was set again and a reminder was send out via email after two weeks.

## Results

### Characteristics of expert panel

297 international experts were invited to participate in the first round of the web-based survey. 105 experts participated in this round (response rate 35%). 53.3% (N = 56) were male. The mean age was 51.3 years and mean work experience was 21.9 years. 49.2% (N = 52) were researchers, 41.9% (N = 44) patient representatives, 12.4% (N = 13) clinicians and 5.7% (N = 6) quality managers (multiple answers possible) total 71 experts of the 105 from the first round responded to the second round (response rate 67.6%). Subgroup analyses of the response rates of the first round revealed that 39% of invited researchers, 34% of invited patient representatives, 35% of invited quality managers and 52% of invited clinicians replied. Further information on the experts’ characteristics based on the first round answers are summarized in Table 2.

**Table 2:** Characteristics of expert panel.

	Round 1 (N = 105)		Round 2 (N = 71)	
	N	in %	N	in %
*Sex*				
Male	56	53.3	37	52.1
Female	49	46.7	34	47.9
*Age*, *years*				
Mean (SD, range)	51.3 (11.9, 22–82)		51.97 (11.7, 27–82)	
*Country of residence*				
Germany	75	71.4	46	64.8
US	12	11.4	9	12.7
UK	3	2.9	3	4.2
Australia	3	2.9	3	4.2
Other countries*	12	11.4	10	14.1
*Professional background (multiple answers possible)*				
Medicine	34	32.4	25	35.2
Psychology	26	24.8	20	28.2
Public health	17	16.2	11	15.5
Politics	6	5.7	4	5.6
Sociology	5	4.8	3	4.2
Other background**	48	44.8	29	40.8
*Stakeholder group (multiple answers possible)*				
Researchers	52	49.52	32	45.1
Patient representatives	44	41.90	22	31.0
Cinicians	13	12.38	10	14.1
Quality managers	6	5.71	6	8.5
*Professional experience*, *years*				
Mean (SD, range)	21.9 (11.0, 2–57)		22.86 (11.25, 3–57)	
*Information on topic* ***				
(1) Not at all well	0	0	-	-
(2) Somewhat well	1	1	-	-
(3) Moderately well	6	5.7	-	-
(4) Well	51	48.8	-	-
(5) Very well	47	44.8	-	-

* (Sweden, Spain, Austria, Italy, Japan, Netherlands, Norway, Canada)

**(e.g. nursing, health services research, education, law, pedagogic)

*** information only available for the first round.

### Results of round 1

Table 3 displays the results of the first round of the experts’ rating on the two criteria relevance and clarity. Regarding the assessment of relevance, the dimensions *patient as unique person*, *patient information* and *clinician-patient communication* received the highest possible median of 9. The two dimensions *integration of medical and non-medical care* and *involvement of family and friends* had the lowest median of 6. Mean values ranged from 5.9 (SD = 2.1) for *integration of medical and non-medical care* to 8.2 (SD = 1.1) for *patient as unique person*. All dimensions reached a consensus ≥ 50% for the tertile seven to nine along the nine-point-scale, except the dimensions *integration of medical and non-medical* and *involvement of family and friends*.

**Table 3:** Results for the relevance (R) and *clarity* (*C*) of the dimensions (N = 105) rating of round 1.

	Relevance							Clarity						
Dimension	Range	Median	Mean	SD	Distribution of ratings (%)*			Range	Median	Mean	SD	Distribution ofratings (%)*		
					1–3	4–6	7–9					1–3	4–6	7–9
*Patient as a unique person*	5–9	9	8.24	1.06	-	5.7	94.3	1–9	7	6.21	1.89	10.5	39.0	50.5
*Biopsychosocial perspective*	3–9	7	7.45	1.53	1.9	18.1	80.0	1–9	5	5.69	1.96	20.0	41.0	39.0
*Essential characteristics of clinician*	4–9	8	7.69	1.35	-	16.2	83.8	3–9	7	6.28	1.85	11.4	35.2	53.3
*Patient involvement in care*	3–9	8	7.78	1.43	1	16.2	82.9	1–9	7	6.45	1.92	11.4	30.5	58.1
*Involvement of family and friends*	2–9	6	6.27	1.80	7.6	45.7	46.7	3–9	7	6.26	1.87	11.4	37.1	51.4
*Physical support*	3–9	7	7.06	1.87	4.8	25.7	69.5	2–9	7	6.30	1.73	12.4	30.5	57.1
*Emotional support*	3–9	7	7.51	1.38	1.9	12.4	85.7	1–9	7	6.21	1.85	9.5	39.0	51.4
*Patient information*	4–9	9	7.97	1.24	-	8.6	91.4	1–9	7	6.71	1.79	4.8	32.4	62.9
*Patient empowerment*	5–9	8	7.55	1.30	-	18.1	81.9	1–9	6	6.10	1.92	14.3	36.2	49.5
*Clinician-patient relationship*	3–9	8	7.57	1.50	1.9	14.3	83.8	1–9	6	6.12	1.87	11.4	41.0	47.6
*Access to care*	2–9	7	6.94	1.82	6.7	25.7	67.6	1–9	6	5.81	1.92	17.1	41.0	41.9
*Integration of medical and non-medical care*	1–9	6	5.90	2.06	17.1	37.1	45.7	1–9	5	5.17	2.31	31.4	32.4	36.2
*Coordination and continuity of care*	3–9	7	7.16	1.64	3.8	21.9	74.3	1–9	7	6.31	1.71	5.7	41.9	52.4
*Teamwork and teambuilding*	1–9	7	6.66	2.08	11.4	21.9	66.7	1–9	6	5.78	2.10	17.1	39.0	43.8
*Clinician-patient communication*	3–9	9	7.92	1.31	1.0	11.4	87.6	1–9	7	6.61	2.02	19.5	30.5	60.0

* Distribution of ratings (%) of the tertiles 1 to 3, 4 to 6 and 7 to 9 along the 9-point-rating-scale.

Regarding the assessment of clarity, a median of 7 was found for nine dimensions, including the dimensions that scored highest in the rating regarding relevance. Four dimensions reached a median of 6 and the two dimensions *biopsychosocial perspective* and *integration of medical and non-medical care* a median of 5. The highest mean was found for the dimension *patient information* (mean = 6.7; SD = 1.8) and the lowest for *integration of medical and non-medical care* (mean = 5.2; SD = 2.3). The distribution of the ratings along the scale for the tertile from seven to nine varied from 62.9% for *patient information* to 36.2% for *integration of medical and non-medical care*. Nine dimensions reached a consensus ≥ 50% for the tertile seven to nine; for six dimensions no consensus was found regarding the distributions on the tertiles.

Regarding the ranking of the five most important dimensions, the following order was found: 1. *patient as a unique person* (rated by 81.9% (N = 86) of the experts on rank one to five); 2. *patient involvement in care* (rated by 54.3% (N = 57) of the experts on rank one to five); 3. *clinician-patient communication* (rated by 52.4% (N = 55) of the experts on rank one to five); 4. *patient information* (rated by 46.7% (N = 49) of the experts on rank one to five); 5. *patient empowerment* (rated by 43.8% N = 46 of the experts on rank one to five). The results are presented in detail in Table A in S1 File. The experts had the opportunity to add further dimensions of patient-centeredness that were missing in their opinion. This question was not mandatory and 23 experts responded to it. The given responses were heterogeneous. Statements that were mentioned only once included for example “empathy of the physician”, “patient competency”, and “patients’ feedback about quality of the treatment”. Two experts explicitly stated that they cannot think of any dimensions that are missing. Two experts advised to include “self-help” as a further independent dimension and another expert suggested including “self-help” into the dimension *emotional support*. Since “self-help” was the only dimension that was proposed by more than one expert, we included “self-help” in the second round. Experts in the second round were asked to rate the relevance and clarity of this dimension. Since only two experts suggested this dimension, we also asked whether or not the experts think that *self-help* should be an independent dimension in the model of patient-centeredness.

### Results of round 2

The experts’ ratings of relevance and clarity given in the second round are presented in Table 4. Most ratings were similar to the first round. The four dimensions *patient as unique person*, *patient information*, *clinician-patient communication*, and *patient involvement in care* were found to be the dimensions with the highest median of 9 regarding the rating of relevance. *Integration of medical and non-medical care* again received the lowest rating of the 15 original dimensions with a median of 6 (mean = 6.1; SD = 1.7). Similar to the first round, the highest mean was found for the dimension *patient as unique person* (mean = 8.5; SD = 0.7). The newly proposed dimension *self-help* reached a median of 7 (mean = 6.3; SD = 2.1). Compared to the first round, the overall ratings slightly increased.

**Table 4:** Results for the relevance and *clarity* (*italic type*) of the dimensions (N = 71) of round 2.

	Relevance							Clarity						
Dimension	Range	Median	Mean	SD	Distribution of ratings (%)*			Range	Median	Mean	SD	Distribution of ratings (%)*		
					1–3	4–6	7–9					1–3	4–6	7–9
*Patient as a unique person*	7–9	9	8.49	.73	-	-	100.0	1–9	7	6.68	1.47	2.8	35.2	62.0
*Biopsychosocial perspective*	3–9	7	7.54	1.33	1.4	14.1	84.5	1–9	6	5.86	1.75	14.1	45.1	40.8
*Essential characteristics of clinician*	4–9	8	7.85	1.31	-	14.1	85.9	3–9	7	6.52	1.50	5.6	31.0	63.4
*Patient involvement in care*	3–9	9	7.97	1.41	1	14.1	84.5	3–9	7	6.73	1.51	2.8	35.2	62.0
*Involvement of family and friends*	3–9	7	6.37	1.71	7.0	42.3	50.7	3–9	7	6.51	1.51	2.8	40.8	56.3
*Physical support*	3–9	7	7.25	1.44	1.4	23.9	74.6	3–9	7	6.66	1.34	2.8	33.8	63.4
*Emotional support*	4–9	8	7.58	1.28	-	12.7	87.3	1–9	7	6.58	1.54	5.6	26.8	67.6
*Patient information*	4–9	9	8.10	1.22	-	7.0	93.0	1–9	7	7.30	1.49	1.4	21.1	77.5
*Patient empowerment*	5–9	8	7.70	1.25	-	16.9	83.1	1–9	7	6.32	1.52	4.2	43.7	52.1
*Clinician-patient relationship*	3–9	8	7.79	1.31	1.4	12.7	85.9	1–9	7	6.35	1.62	5.6	39.4	54.9
*Access to care*	3–9	7	7.24	1.64	1.4	29.6	69.0	1–9	6	6.21	1.67	9.9	40.8	49.3
*Integration of medical and non-medical care*	2–9	6	6.11	1.70	9.9	40.8	49.3	1–9	5	5.27	1.83	18.3	52.1	29.6
*Coordination and continuity of care*	3–9	7	7.30	1.53	2.8	21.1	76.1	1–9	7	6.54	1.53	2.8	39.4	57.7
*Teamwork and teambuilding*	1–9	7	6.86	1.80	5.6	28.2	66.2	1–9	6	6.13	1.80	11.3	40.8	47.9
*Clinician-patient communication*	5–9	9	8.28	1.20	-	8.5	91.5	3–9	7	7.15	1.67	4.2	23.9	71.8

* Distribution of ratings (%) of the tertiles 1 to 3, 4 to 6 and 7 to 9 along the 9-point-rating-scale.

The distribution of the ratings along the nine-point rating scale became narrower, indicating that the consensus increased. Fourteen dimensions reached a consensus ≥ 50% for the tertile seven to 9, and within these, nine dimensions even reached a consensus of ≥ 80%. For one dimension (*integration of medical and non-medical care*) the consensus on the tertile seven to nine was slightly below 50%, namely 49.3%.

Regarding the criterion of clarity, the results were similar to the results of the first round. Eleven dimensions had a median of 7. Three dimensions reached a median of 6. The dimension with the lowest perceived clarity was again *integration of medical and non-medical care* (median = 5; mean = 5.3; SD = 1.8). Only the newly proposed dimension *self-help* was rated lower (median = 5; mean = 5.2; SD = 2.3). Again, the highest mean was found for the dimension *patient information* (mean = 7.3; SD = 1.5). As in round one, consensus was lower for the rating of clarity than for the rating of relevance. Eleven dimensions reached a consensus ≥ 50% for the tertile seven to nine. For two dimensions the consensus on the tertile seven to nine was slightly below 50%, namely 49.3% for *access to care* and 47.9% for *teamwork and teambuilding*. For the dimensions *integration of medical and non-medical care*, *biopsychosocial perspective*, as well as for the new dimension *self-help* no consensus was found. Therefore, not all dimensions fulfilled the pre-defined threshold for the validation of the model. The dimension *integration of medical and non-medical care* failed to reach a median ≥ 7 on both criteria. Consequently it did not achieve a consensus ≥ 50% on the seven to nine tertile, and therefore did not reach sufficient rating for the validation of our model. The three dimensions *access to care*, *teamwork and teambuilding*, and *biopsychosocial perspective* did reach a median ≥ 7 regarding relevance, but only a median of 6 regarding clarity. Thereby these dimensions also failed our threshold for validation.

The order of the priority ranking of the five most important dimensions hardly changed during the second round. Only *patient information* was now rated third (rated by 54.9% (N = 39) of the experts on rank one to five) and *clinician-patient communication* in fourth position (rated by 53.5% (N = 55) of the experts on rank one to five). The results can be found in detail in the Table B in S1 File.

The majority of experts (63.4%) were against including the newly proposed dimension *self-help* as a new independent dimension in the model of patient-centeredness.

Finally, subgroup analyses of the results of the second round are shown in detail in Table A and Table B in S2 File. For the criterion relevance, differences of at least 1.5 points on the rating scale were found for the following dimensions: *essential characteristics of clinician* (lowest median in subsample of clinicians with 7.5, highest median in subsample of patient representatives with 9), *involvement of family and friends* (lowest median in subsamples of clinicians and quality managers with 5.5, highest median in subsample of researchers with 7), *access to care* (lowest median in subsamples of clinicians and quality managers with 5.5, highest median in subsample of researchers with 7) and *integration of medical and non-medical care* (lowest median in subsamples of researchers and clinicians with 5, highest median in subsample of patient representatives with 7).

On the criterion clarity, the only difference of ≥ 1.5 points between subgroups was found for *access to care* (lowest median in subsample of quality managers with 5, highest median in subsample of patient representatives with 7). Due to small sample sizes of certain subgroups, no significance testing was conducted. *Self-help* was not considered in this analysis since the majority of the stakeholders did not rate it as an independent dimension.

### Model of patient-centered care

**Fig 1:**
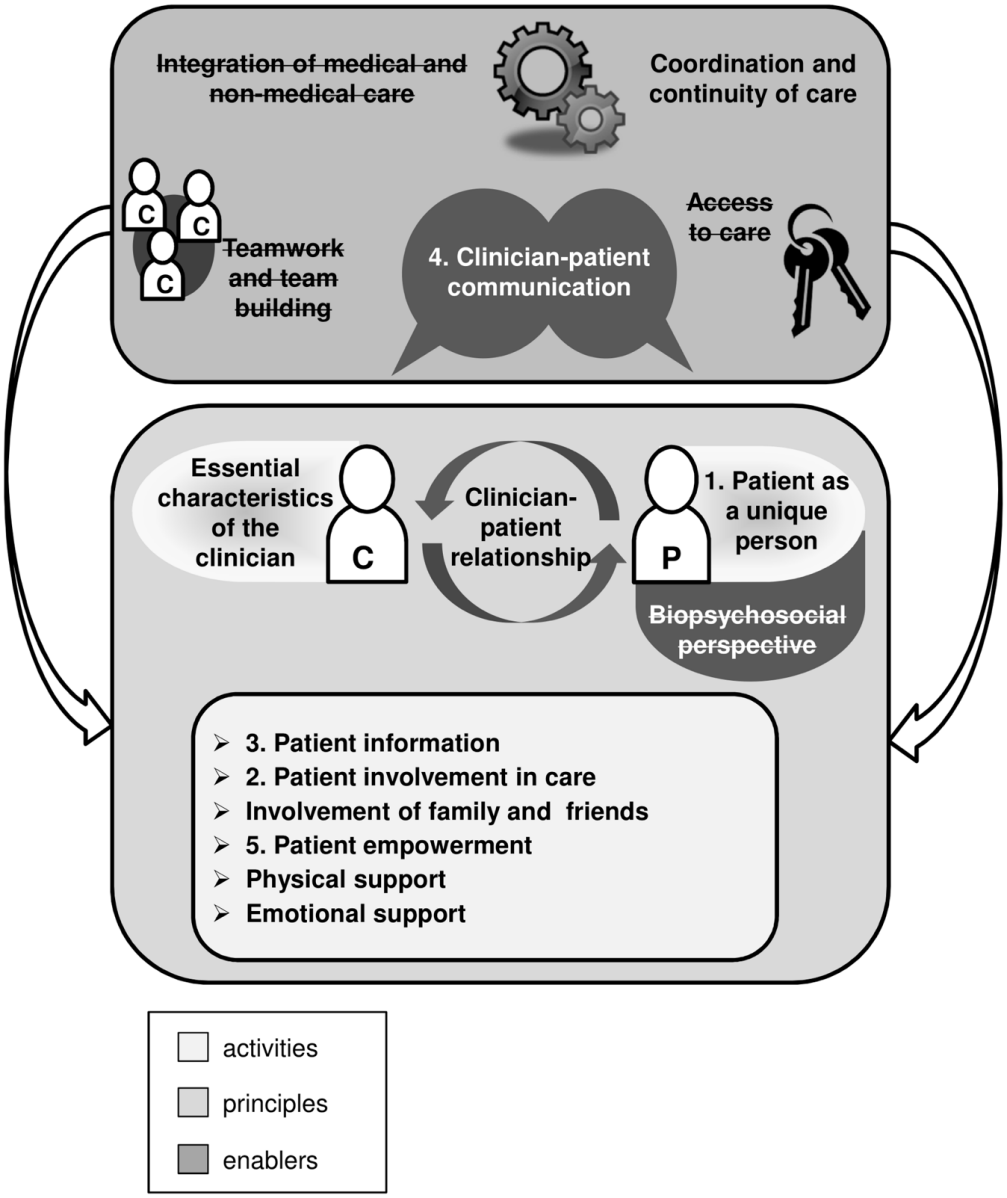
Model of patient-centeredness [23]. The dimensions in the upper square presenting the *enablers* of patient-centeredness. I In the lower square the *principles* are shown, which frame the *activities* of patient-centeredness. The dimensions rated as the top five out of the fifteen dimensions are labeled with the numbers of their ranking of importance. The four dimensions that were rated with a median < 7 on at least one of the criteria (relevance or clarity) are crossed out.

Based on the results of the Delphi survey, we present a revision of the original model including the relevance and clarity rating of the experts in Fig 1. The original model was developed based on a systematic review [22], which was conducted prior to this Delphi study. In this model we aligned the 15 dimensions to three categories namely a) *principles*, b) *enablers*, and c) *activities*. This proposed differentiation showed the interrelation of the dimensions. We found the dimensions *patient as a unique person*, *biopsychosocial perspective*, *essential characteristics of the clinician* and *clinician-patient relationship* as the underlying *principles* of a patient-centered care. For the implementation of these principles the patient-centered *activities*, i.e. *patient information*, *patient involvement in care*, *involvement of family and friends*, *patient empowerment*, *physical* and *emotional support* are needed. Patient-centered care can be further facilitated and implemented through certain enablers that we found in the dimensions *continuity of care*, *clinician-patient communication*, *integration of medical and non-medical care*, *teamwork and team building* and *access to care*.

Three of the five dimensions that were named *enablers* of patient-centered care were rated as insufficient relevant and/or clear in the proposed model. However, one of the *enablers*, namely *physician-patient communication* has been rated as one of the five most important dimensions. From the four dimensions that were labeled as *principles* of patient-centeredness, *patient as a unique person* was rated as the most important dimension and the dimension *biopsychosocial perspective* was judged to be insufficiently clear. Those dimensions described as patient-centered *activities* all reached sufficiently high ratings and three were considered in the top five rating of importance.

## Discussion

### Principal findings

This study was based on a previous systematic review, which identified 15 dimensions related to patient-centeredness that were summarized into an integrative model [23], describing *enablers*, *principles* and *activities* of patient-centered care. The Delphi process aimed to validate the dimensions of this model using experts’ ratings. Furthermore, the aim of this study was to increase the consensus regarding the relevance and clarity of the dimensions and to prioritize the dimensions.

The results of the second round of the Delphi procedure were used for the final validation of the proposed model of patient-centeredness. In this second round, eleven dimensions reached a median ≥ 7 on both of the criteria. Three dimensions achieved a median ≥ 7 regarding relevance but not regarding clarity. The comparison of the results between the first and the second round revealed that the relevance and clarity ratings as well as the overall consensus increased from the first to the second round. One dimension, namely *integration of medical and non-medical care* reached a median < 7 only on the both criteria. Therefore this dimension was clearly not sufficiently validated according to our criteria. However, the subgroup analysis showed that this dimension was mainly rated as insufficient by researchers and clinicians of the panel and received higher ratings from the patient representatives. Accordingly, we suggest further research on the relevance and clarity of this dimension and point out that certain dimensions of patient-centered care might be of different importance to different stakeholder groups.

The dimensions *biopsychosocial perspective*, *access to care* and *teamwork and teambuilding* were also not rated sufficiently high to reach our validation threshold. However, these dimensions were rated as very relevant and only their ratings on clarity were below seven. Furthermore, the dimensions *biopsychosocial perspective* and *access to care* are included in very prominent models of patient-centeredness [16, 19, 20] and we strongly recommend further research on their content to allow clearer descriptions.

This can be equally advised for the other dimensions. Ratings regarding clarity were mostly below the ratings regarding relevance and ranged from *moderately* to *very clear*. The results of this study support the impression gained by our prior systematic literature review [23], i.e. that although the topic is highly relevant, there exists uncertainty about its content.

We could clearly identify five dimensions that were rated as the most important by the experts. Not surprisingly, some of these dimensions appear in other well established models. For example, the dimension *patient as a unique person* overlaps with the dimensions „patient-as a-person”in the model of Mead and Bower (20] and the element “exploring both the disease and the illness experience” in the definition of Stewart (17]. The dimension *patient involvement in care* is included in Stewarts’ et al. [17] dimension “finding common ground” and Epsteins [12] aspect “helping patients to share power and responsibility by involving them in choices to the degree that they wish”.

From the experts’ viewpoint, the proposed model seems to be complete and consequently no new dimension was included into the model.

### Strengths and limitations

The present study has several strengths. First of all, the model validated in the Delphi survey was based on a large body of literature [23]. Second, stakeholders from twelve different countries participated in the study leading to a broad range of judgments, although they mainly reflect opinions from western countries. Third, different stakeholder groups were included. Almost half of the panel members were patient representatives. This is a major advantage of our study, as to our knowledge, the views of patients or patient representatives have not been included sufficiently in the prior development of definitions and models on patient-centeredness.

There are also several limitations of this study. First of all, the overall response rate was rather low with 35%. Of the n = 297 international experts, who were invited to participate in the Delphi survey, n = 105 responded to the first round and n = 71 of the n = 105 took part in the second round. Other studies including clinical experts achieved similar response rates [28, 29]. A possible explanation for the partially low response rates could be that the invited experts assumed that participating in a two-step Delphi survey would be too time-consuming. Moreover, we received several emails from invited experts apologizing for missing the deadline and stating that they would have liked to participate. Therefore, the timeframe for participation was possibly too short. Second, groups of experts were not distributed equally. The sample was mainly represented through researchers (49%) and patient-representatives (41%). Less quality managers and clinicians were invited in the first place since the authors identified fewer contacts in this field. Third, due to the heterogeneous sizes of stakeholder groups, differences between the groups could not be interpreted meaningfully. Especially, for the dimensions *involvement of family and friends*, *access to care* and *integration of medical and non-medical care* ratings differed for the subgroups. Therefore, we strongly recommend taking different stakeholders’ opinions into account when conducting further research. However, especially for the five dimensions rated as most important, consensus for both criteria was rather high. Fourth, despite of having included patient representatives, we did not include patients in our study due to several reasons. The methods we used for the model development, namely a systematic review [23] and the Delphi procedure set a very academic framework to our study. This can be seen as an advantage on the one hand, however, it limited the possibility to include patients. It would have been necessary to include more detailed instructions and guidance as it was possible in our study. Therefore we decided to include patient representatives instead. As we were able to include 44 patient representatives, we think that we did reach the goal to include the patients’ point of view, although yet only from a more academic level. It would be a further step to continue the validation of this model with real patients using a qualitative approach, e.g. focus groups or interviews.

### Implications for practice and further research

A concise definition of patient-centeredness is the precondition for the implementation of the concept in routine care. The proposed model provides a framework with 15 dimensions comprising current definitions of patient-centeredness. These dimensions have now been validated from an experts’ viewpoint, including researchers, clinicians, quality managers and patient representatives. Therefore our model gives a further direction towards the definition that is urgently needed to implement the concept of patient-centeredness in routine care [1, 11, 30]. The model can be used as an orientation for the development of new medical guidelines, health policy definitions, as well as in curricula of health care and medical education, which focus on the concept of patient-centeredness. It provides a foundation for researchers, clinicians, policy makers and patients (representatives) to speak the same language and it fastens the implementation process if all concerned parties are pulling in the same direction.

However, as stated in the limitations we are still a step ahead from a final definition of patient-centeredness. Although most dimensions are considered to be relevant, this study highlighted that more research on the clarity of the content of some of the dimensions is required. Therefore, we will conduct an expert workshop with the participation of the same stakeholder groups that participated in this Delphi study [22]. Within this workshop we will further discuss the improvement of the content of the identified dimensions. Moreover it will be indispensable to validate the model from a patients’ viewpoint and focus on the inclusion of real patients in a further study.

Furthermore, the results of this study indicate the five most important dimensions. The focus of future research should be directed towards these dimensions. In order to identify missing gaps in the measurement of patient-centeredness, the authors suggest to examine the operationalization of measures of these dimensions and to find evidence for its effectiveness [22]. This has been partly done in systematic reviews on the measurement of some of the dimensions that have been rated as most important in this study, namely *patient involvement in care* [31], *patient empowerment* [32], *physician-patient communication* [33] and *clinician-patient relationship [34].*

Additional challenges for further research resulting from this study will be to adapt this general model for different health care systems and settings (e.g. in an outpatient versus an inpatient setting), including a possible adaptation to fit non-western cultural settings.

## Conclusion

This study provides a validated model of patient-centeredness that can be used in studies on measurement, effectiveness and implementation of the concept, as well as in clinical settings of modern health care. Additionally, it can be applied in health education curricula and the development of policy guidelines. Moreover, this study highlights current research gaps regarding the definition of patient-centeredness and leads a direction towards more research on this field.

## Supporting Information

S1 File Including Table A in S1 File Priority ranking of round 1 and Table B in S1 File Priority ranking of round 2 (DOCX)

S2 File Including Table A in S1 File Results (median, mean (SD)) for stakeholder groups (N = 81, multiple allocation) in round 2 for the criterion relevance and Table B in S1 File Results (median, mean (SD)) for stakeholder groups in round 2 for the criterion clarity (DOCX)
