# Supplementary material for: Which Dimensions of Patient-Centeredness Matter? - Results of a Web-Based Expert Delphi Survey
Source: PLoS One. 2015 Nov 5;10(11):e0141978. doi: 10.1371/journal.pone.0141978 (PMC4635011; doi:10.1371/journal.pone.0141978)
Supplement: S1 File — (DOCX) [file pone.0141978.s001.docx]

**S1 File**

**Table A** Priority ranking of round 1 in %(N).

| Dimension | 1. | 2. | 3. | 4. | 5. | Ranking 1 to 5 | No Ranking* |
| --- | --- | --- | --- | --- | --- | --- | --- |
| *Patient as a unique person* | 62.9 (66) | 10.5 (11) | 4.8 (5) | 2.9 (3) | 1.0 (1) | 81.9 (86) | 18.1 (19) |
| *Biopsychosocial perspective* | 5.7 (6) | 10.5 (11) | 8.6 (9) | 6.7 (7) | 3.8 (4) | 35.2 (37) | 64.8 (68) |
| *Essential characteristics of clinician* | - | 6.7 (7) | 3.8 (4) | 3.8 (4) | 5.7 (6) | 21.0 (20) | 80.0 (84) |
| *Patient involvement in care* | 5.7 (6) | 16.2 (17) | 13.3 (14) | 7.6 (8) | 11.4 (12) | 54.3 (57) | 45.7 (48) |
| *Involvement of family and friends* | - | 1.9 (2) | 2.9 (3) | 1.9 (2) | 6.7 (7) | 13.3 (14) | 86.7 (91) |
| *Physical support* | - | 2.9 (3) | 1.0 (1) | 4.9 (5) | 1.0 (1) | 9.5 (10) | 90.5 (95) |
| *Emotional support* | 1.0 (1) | 2.9 (3) | 6.7 (7) | 3.8 (4) | 4.8 (5) | 19.0 (20) | 81.0 (85) |
| *Patient information* | 5.7 (6) | 7.6 (8) | 16.2 (17) | 10.5 (11) | 6.7 (7) | 46.7 (49) | 53.3 (56) |
| *Patient empowerment* | 1.9 (2) | 6.7 (7) | 13.3 (14) | 14.3 (15) | 7.6 (8) | 43.8 (46) | 56.2 (59) |
| *Clinician-patient relationship* | 2.9 (3) | 9.5 (10) | 6.7 (7) | 13.3 (14) | 6.7 (7) | 39.0 (41) | 61.0 (64) |
| *Access to care* | 3.8 (4) | 3.8 (4) | 3.8 (4) | 2.9 (3) | 2.9 (3) | 17.1 (18) | 82.9 (87) |
| *Integration of medical and non-medical care* | - | 1.0 (1) | 1.0 (1) | 2.9 (3) | 2.9 (3) | 7.6 (8) | 92.4 (97) |
| *Coordination and continuity of care* | - | 1.0 (1) | 5.7 (6) | 4.8 (5) | 16.2 (17) | 27.6 (29) | 72.4 (76) |
| *Teamwork and teambuilding* | - | - | 1.9 (2) | 3.8 (4) | 5.7 (6) | 11.4 (12) | 88.6 (93) |
| *Clinician-patient communication* | 7.6 (8) | 15.2 (16) | 6.7 (7) | 12.4 (13) | 10.5 (11) | 52.4 (55) | - 1. (50) |

*% (N) of the experts that did not rate this dimension for the five most important

**Table B** Priority ranking of round 2 in %(N).

| Dimension | 1. | 2. | 3. | 4. | 5. | Ranking 1 to 5 | No Ranking |
| --- | --- | --- | --- | --- | --- | --- | --- |
| *Patient as a unique person* | 73.2 (52) | 7.0 (5) | 4.2 (3) | - | 1.4 (1) | 85.9 (61) | 14.1 (10) |
| *Biopsychosocial perspective* | 2.8 (2) | 9.9 (7) | 5.6 (4) | 7.0 (5) | 4.2 (3) | 29.6 (21) | 70.4 (50) |
| *Essential characteristics of clinician* | - | 5.6 (4) | 4.2 (3) | 1.4 (1) | 4.2 (3) | 15.5 (11) | 84.5 (60) |
| *Patient involvement in care* | 4.2 (3) | 31.1 (22) | 12.7 (9) | 7.0 (5) | 9.9 (7) | 64.8 (46) | 35.2 (25) |
| *Involvement of family and friends* | - | 1.4 (1) | - | 2.8 (2) | 7.0 (5) | 11.3 (8) | 86.7 (91) |
| *Physical support* | 1.4 (1) | - | 1.4 (1) | 1.4 (1) | - | 4.2 (3) | 95.8 (68) |
| *Emotional support* | - | 4.2 (3) | 5.6 (4) | 4.2 (3) | 2.8 (2) | 16.9 (12) | 83.1 (59) |
| *Patient information* | 1.4 (1) | 8.5 (6) | 15.5(17) | 22.5 (16) | 7.0 (5) | 54.9 (39) | 45.1 (32) |
| *Patient empowerment* | 1.4 (1) | 5.6 (4) | 15.5(17) | 8.5 (6) | 19.7 (14) | 50.7 (36) | 49.3 (35) |
| *Clinician-patient relationship* | 2.8 (2) | 12.7 (7) | 12.7 (7) | 14.1 (10) | 4.2 (3) | 46.5 (33) | 53.5 (38) |
| *Access to care* | 4.2 (3) |  | 2.8 (2) | 1.4 (1) | 5.6 (4) | 14.1 (10) | 85.9 (61) |
| *Integration of medical and non-medical care* | - | - | 1.4 (1) | 2.8 (2) | - | 4.2 (3) | 95.8 (68) |
| *Coordination and continuity of care* | 1.4 (1) | 2.8 (2) | 1.4 (1) | 2.8 (2) | 14.1 (10) | 2256 (16) | 77.5 (55) |
| *Teamwork and teambuilding* | - | 1.4 (1) | - | 1.4 (1) | 5.6 (4) | 8.5 (6) | 91.5 (65) |
| *Clinician-patient communication* | 4.2 (3) | 7.0 (5) | 14.1 (10) | 19.7 (14) | 8.5 (6) | 53.5 (55) | 46.5 (33) |

*% (N) of the experts that did not rate this dimension for the five most important
