# Supplementary material for: Which Dimensions of Patient-Centeredness Matter? - Results of a Web-Based Expert Delphi Survey
Source: PLoS One. 2015 Nov 5;10(11):e0141978. doi: 10.1371/journal.pone.0141978 (PMC4635011; doi:10.1371/journal.pone.0141978)
Supplement: S2 File — (DOCX) [file pone.0141978.s002.docx]

**S2 File**

**Table A Results (median, mean (SD)) for stakeholder groups (N=81, multiple allocation).in round 2 for the criterion relevance**

| Dimension | Researcher (N=43) | Patient representatives (N=22) | Clinicians (N=10) | Quality managers (N=6) |
| --- | --- | --- | --- | --- |
| *Patient as a unique person* | 9, 8.49 (.74) | 9, 8.59 (.74) | 8.5, 8.3 (.82) | 8.50, 8.33 (.82) |
| *Biopsychosocial perspective* | 7, 7.49 (1.32) | 8, 7.64 (1.43) | 7, 7.20 (.79) | 7, 7.5, (1.23) |
| *Essential characteristics of clinician* | 8, 7.67 (1.36) | 9, 8.14 (1.28) | 7.5, 7.3 (.95) | 8, 8.17 (.75) |
| *Patient involvement in care* | 9, 8.19 (1.18) | 8, 7.68 (1.72) | 8, 7.7 (1.34) | 8.50, 7.83 (1.47) |
| *Involvement of family and friends* | 7, 6.44 (1.71) | 6, 6.32 (1.75) | 5.5, 5.5 (1.35) | 5.5, 5.67 (2.16) |
| *Physical support* | 7, 7 (1.07) | 8, 7.82 (1.10) | 7, 7.2 (1.48) | 7, 7.17 (.75) |
| *Emotional support* | 7, 7.51 (1.36) | 8, 7.73 (1.32) | 7, 7.3 (.48) | 8, 7.8, (.75) |
| *Patient information* | 9, 8.19 (1.10) | 9, 8.05 (1.50) | 8.5, 8.3 (.95) | 8, 7.83 (1.17) |
| *Patient empowerment* | 8, 7.67 (1.25) | 8, 7.68 (1.32) | 8, 7.6 (1.17) | 8, 8, (1.10) |
| *Clinician-patient relationship* | 8, 7.74 (1.38) | 8, 7.95 (1.25) | 8, 7.9 (.99) | 8, 8, (.89) |
| *Access to care* | 7, 6.81 (1.57) | 9, 8.32 (1.04) | 6, 6.10 (1.97) | 6.5, 6.83, (1.83) |
| *Integration of medical and non-medical care* | 5, 5.12 (1.71) | 7, 7 (1.66) | 5, 5 (1.41) | 5.5, 5.83 (2.04) |
| *Coordination and continuity of care* | 7, 7.33 (1.67) | 7, 7.23 (1.31) | 7.5, 7.6 (1.27) | 7.5, 7.67 (1.21) |
| *Teamwork and teambuilding* | 7, 6.52 (1.97) | 8, 7.68 (1.71) | 7, 7 (1.70) | 7, 7.33 (.52) |
| *Clinician-patient communication* | 9, 8.12 (1.24) | 9, 8.59 (1.01) | 8.5, 8.4 (.70) | 9, 8.67 (.52) |

**Table B Results (median, mean (SD)) for stakeholder groups in round 2 for the criterion clarity**

| Dimension | Researcher (N=43) | Patient representatives (N=22) | Clinicians (N=10) | Quality managers (N=6) |
| --- | --- | --- | --- | --- |
| *Patient as a unique person* | 7, 6.84 (1.27) | 7, 6.45 (1.96) | 7, 7 (1.25) | 6.50, 6.50 (.54) |
| *Biopsychosocial perspective* | 6, 6.07 (1.55) | 5.50, 5.59 (1.99) | 5.50, 5.4 (1.51) | 6, 5.83 (1.94) |
| *Essential characteristics of clinician* | 7, 6.42 (1.38) | 7, 6.68 (1.72) | 6, 6 (1.49) | 7, 6.33 (1.21) |
| *Patient involvement in care* | 7, 6.98 (1.47) | 6, 6.32 (1.55) | 6.5, 6.3 (1.33) | 7, 6.83 (1.32) |
| *Involvement of family and friends* | 7, 6.72 (1.35) | 6, 6.05 (1.81) | 6.5, 6.5 (1.51) | 6.5, 6.5 (1.51) |
| *Physical support* | 7, 6.58 (1.35) | 8, 7.82 (1.10) | 7, 7.3 (1.16) | 7, 7 (.00) |
| *Emotional support* | 7, 6.63 (1.27) | 7, 6.50 (1.90) | 7, 6.8 (.79) | 7, 6.33 (2.06) |
| *Patient information* | 7, 7.35 (1.25) | 7, 7 (1.98) | 8, 7.8 (1.23) | 8, 7.67 (1.37) |
| *Patient empowerment* | 6, 6.42 (1.35) | 6.50, 6.14 (1.91) | 6.50, 6.10 (1.10) | 6.5, 6 (1.27) |
| *Clinician-patient relationship* | 7, 6.49 (1.45) | 7, 6.32 (1.89) | 6, 5.90 (1.60) | 6, 5.83 (1.84) |
| *Access to care* | 6, 6.26 (1.36) | 7, 6.41 (2.10) | 6, 5.80 (1.55) | 5, 5 (1.90) |
| *Integration of medical and non-medical care* | 5, 5.12 (1.71) | 5, 5.59 (1.94) | 5, 4.60 (1.41) | 4, 5 (2.45) |
| *Coordination and continuity of care* | 7, 6.72 (1.30) | 6.5, 5.95 (1.99) | 7, 7.10 (1.20) | 7, 7 (1.09) |
| *Teamwork and teambuilding* | 7, 6.19 (1.59) | 6.5, 6.09 (2.18) | 6.50, 6.50 (1.78) | 5.50, 5.83 (1.94) |
| *Clinician-patient communication* | 7, 7.14 (1.44) | 7.50, 6.91 (2.20) | 7.50, 7.60 (1.17) | 8, 7.83 (1.17) |
